# ‘A phase II study of oral uracil/ftorafur (UFT®) plus leucovorin combined with oxaliplatin (TEGAFOX) as first-line treatment in patients with metastatic colorectal cancer’

**DOI:** 10.1038/sj.bjc.6602913

**Published:** 2005-12-13

**Authors:** J Bennouna, H Perrier, B Paillot, F Priou, J H Jacob, M Hebbar, S Bordenave, J F Seitz, F Cvitkovic, E Dorval, K Malek, D Tonelli, J Y Douillard

**Affiliations:** 1 Centre René Gauducheau, Nantes, France; 2 Hôpital St Joseph, Marseille, France; 3 CHU Charles Nicolle, Rouen, France; 4 Centre Hospitalier Départemental, La Roche-sur-Yon, France; 5 Centre François Baclesse, Caen, France; 6 Hôpital Claude Huriez, Lille, France; 7 CHU Hôtel Dieu, Nantes, France; 8 Hôpital La Timone Adultes, Marseille, France; 9 Centre René Huguenin, St Cloud, France; 10 Hôpital Trousseau, Tours, France; 11 Bristol-Myers Squibb, Rueil-Malmaison, France

**Keywords:** colorectal cancer, combination treatment, UFT®, oxaliplatin

## Abstract

This phase II trial was performed to evaluate the efficacy and tolerability of a new combination of Uracil/Ftorafur (UFT®)/leucovorin (LV) and oxaliplatin in patients (pts) with metastatic colorectal cancer (MCRC) who had not received prior chemotherapy for metastatic disease. Between February 2002 and October 2002, 64 patients received UFT® 300 mg m^−2^ day^−1^ and LV 90 mg day^−1^ from day 1 to day 14 combined with oxaliplatin 130 mg m^−2^ on day 1, every 3 weeks. All patients were evaluable for safety analysis and 58 of 64 patients were eligible for efficacy. Responses were reviewed by an independent review committee. Of the 58 per-protocol defined assessable patients, 1 complete response and 20 partial responses were observed yielding a response rate of 34% (95% CI: 22–47). The median response duration was 8.74 months (range 1.6–14). The median time to progression and the median survival were 5.88 months (95% CI: 4.34–8.21) and 18.2 months (95% CI: 10–20.7), respectively. Diarrhoea and peripheral neuropathy were the most frequent and predictable toxicities. These events were reversible, noncumulative and manageable. Grade 3 diarrhoea occurred in only 11% of the patients. No grade 4 gastrointestinal toxicity was reported in the study. The incidence of grade 3/4 (National Cancer Institute Common Toxicity Criteria 2: NCI-CTC 2) peripheral neuropathy was 15%. Haematological toxicity was of mild to moderate intensity with 10% of the patients with Grade 3/4 neutropenia without any episode of complication. The TEGAFOX regimen, a new combination using UFT®/LV and oxaliplatin every 3 weeks is feasible on an outpatient basis. The combination is safe and active and may offer a promising alternative to the intravenous route. Nevertheless this efficacy results should be confirmed by randomized phase III trials.

Colorectal cancer represents a significant global public health problem, especially in populations with westernized lifestyles. Colorectal cancer is the fourth commonest cancer worldwide; it accounts for 9.4 and 10.1% of all cancer in men and women (Parkin *et al*, 1998, 1999), respectively.

Following diagnosis, approximately 50% of patients with colorectal cancer subsequently develop locally recurrent or distant metastatic disease. Treatment of patients with metastatic colorectal cancer with fluoropyrimidine therapy is well established; however, initial results with 5-fluorouracil (5-FU) administered as a single agent, by rapid intravenous (i.v.) injection, were disappointing. Consequently, more recent research efforts have concentrated on optimizing the administration of fluoropyrimidine therapy, and investigating 5-FU in combination with other antitumour agents.

Ftorafur, a prodrug of 5-FU, is converted to 5-FU by hepatic metabolism and demonstrates almost complete bioavailability after oral administration (Anttila *et al*, 1983). While ftorafur has shown efficacy in a number of solid tumour types (Ansfield *et al*, 1983; Taguchi, 1997), coadministration with uracil results in significantly increased 5-FU concentration within the tumour site (Nakajima *et al*, 1980, Maeda *et al*, 1981; Suemasu *et al*, 1982; Tsujimoto *et al*, 1983). UFT® is a combination of uracil and ftorafur for oral administration. It was developed as a convenient treatment to avoid the need for repeated i.v. injections of 5-FU. UFT® is available commercially in Japan and Europe.

Two large randomized phase III trials enrolled patients with metastatic colorectal cancer to either UFT® (300 mg m^−2^ day^−1^) plus LV (75 or 90 mg day^−1^) for 28 days every 35 days, or a Mayo Clinic regimen every 28 days (Douillard *et al*, 2002) or 35 days (Carmichael *et al*, 2002). In the largest study (Douillard *et al*, 2002) (*n*=816), the oral UFT®/LV treatment was broadly comparable with the Mayo Clinic regimen with respect to median survival time (12.4 months *vs* 13.6 months, respectively; *P*=0.63) and overall response rate (11.7 *vs* 14.5%, respectively; *P*=0.232). Median time to progression was more favourable for oral UFT®/LV (3.8 months; *P*=0.01) than for i.v. 5-FU/LV (3.5 months). In the second study Carmichael *et al* (2002) (*n*=380), efficacy as assessed by time to progression in the oral UFT®/LV group was comparable with i.v. 5-FU/LV treatment. In both phase III studies, gastrointestinal toxicity, including diarrhoea, stomatitis, nausea, and vomiting, were more frequent with the Mayo Clinic regimen as were hand-foot syndrome and myelotoxicity. Use of antibiotics, growth factors and antiemetics were less frequent with UFT®/leucovorin.

The addition of oxaliplatin to first-line treatment with 5-FU/LV is reported to have significantly increased progression-free survival and improved response rate in patients with advanced colorectal cancer compared with 5-FU/LV alone (de Gramont *et al*, 2000).

Outcomes such as the above provided the motivation for a phase I dose ranging study of UFT®/LV plus oxaliplatin (Douillard and Seitz, 2001). This study evaluated oral UFT® (from 200–350 mg m^−2^ day^−1^) plus i.v. oxaliplatin (single infusion of 100 mg – 130 mg m^−2^ on day 1 of the treatment cycle) and oral LV (fixed dose, 90 mg day^−1^) in 14-day cycles in 21 patients with metastatic colorectal cancer. Although this study was not designed to assess efficacy, a partial response was apparent in five of the 18 evaluable patients (overall response 28%). In this phase I study, the safety was consistent with the tolerability profiles of UFT® and oxaliplatin. Gastrointestinal disturbance was associated with the study treatment combination. Grade 1 or 2 diarrhoea occurred in 43% of patients, grade 3 or 4 in 19% of patients. Nausea/vomiting was mainly of grade 1 or 2, with 10% of patients reporting grade 3 episodes. Neurosensory toxicity occurred in 86% of patients, but was of mild to moderate, only reaching grade 3 in one patient.

The maximum tolerated dose of the combination was established as: UFT® 350 mg m^−2^ day^−1^, LV 90 mg day^−1^, oxaliplatin 130 mg m^−2^, with grade 3 diarrhoea and grade 3 vomiting as the main dose limiting toxicities (DLTs).

Thus, the recommended dose and schedule for further phase II trial was UFT 300 mg m^−2^ day^−1^ plus leucovorin 90 mg daily, on days 1–14, and i.v. oxaliplatin 130 mg m^−2^ on day 1 every 3 weeks.

Given the results of this phase I trial, we initiated a phase II study using this regimen to determine the efficacy and safety of the combination as first-line treatment in metastatic colorectal cancer patients

## PATIENTS AND METHODS

### Patient selection

Male and female patients, age 18–75 years, with histologically or cytologically confirmed colorectal adenocarcinoma not suitable for curative surgery, with at least one uni or bidimensionally measurable target lesion in a nonirradiated area were included in this multicentre, phase II, open-label study. Further eligibility criteria included the following: Eastern Cooperative Oncology Group (ECOG) performance status of 0 or 1; adequate bone marrow reserve (absolute neutrophils⩾2000 mm^3^ and platelets >125 000 mm^−3^), adequate liver (serum bilirubin⩽1.5 times the upper limits of normal (ULN), AST and ALT⩽2.5 times ULN or ⩽5 times ULN in case of hepatic metastasis) and renal (serum creatinine⩽1.5 times ULN) functions.

Patients were excluded if they had received previous chemotherapy for metastatic disease, or had brain metastases or a history of other neoplasms (except nonmelanoma skin carcinoma or cured carcinoma *in situ* of the cervix), a serious active infection or other underlying condition that would impair the patient from receiving study medication, or peripheral neuropathy ⩾grade 1 NCI-CTC 2. Females of childbearing potential had to have a negative serum or urine pregnancy test within 72 h of starting study medication and had to use adequate contraceptive measures during the study. Pregnant or lactating women were excluded. Prior adjuvant or neo-adjuvant therapy was allowed, if stopped at least 4 weeks before study entry, and providing patients had recovered from the effects of previous chemotherapy.

Written informed consent was obtained from each participating patient before entry into study. The study protocol was reviewed on 08 January 2002 by the French Ethics Committee (CCPPRB) of Region des Pays de Loire, Nantes, France. The study was designed according to the current revision of the Declaration of Helsinki and conducted in accordance with good clinical practice.

### Treatment plan and dose modifications guidelines

#### Treatment administration

UFT® and LV were taken orally in three divided doses, on a daily basis (i.e. every 8 h) on days 1–14 of the cycle at the dose of 300 mg m^−2^ day^−1^ and 90 mg day^−1^, respectively. The food was not consumed for 1 h before and after UFT® administration. Since the available capsule strength of UFT® is 100 mg (based ftorafur), the total daily dosage was rounded up or down to the nearest 100 mg.

If UFT® was withheld, LV was also omitted. Oxaliplatin was given at the dose of 130 mg m^−2^, as a 2-h i.v. infusion in dextrose 5%, on day 1 of each cycle.

Doses of UFT ®and oxaliplatin were based on body surface area up to a maximum of 2.0 m^2^; thus the total dose per administration did not exceed 600 mg for UFT and 260 mg for oxaliplatin. Treatment was administered on an outpatient basis every 3 weeks and consisted of at least two cycles. Duration of treatment was based on tumour response: patients with stable disease (SD) or partial response (PR) could receive treatment until progression, those with complete response (CR) up to four cycles after CR. Patients were taken off study at any time if progression or unacceptable toxicity occurred.

#### Dose modification

Dose reductions for both UFT® and oxaliplatin were based on toxicity from the previous cycle and graded according to NCI-CTC 2 classification. Up to two dose reductions for UFT® (by 50 mg m^−2^ day^−1^) and oxaliplatin (by 15 mg m^−2^) were allowed. Re-escalation of dosage after a reduction was not permitted. Doses of oxaliplatin were reduced by one dose level for persistent paresthesia (>18 days) and paresthesia with pain/functional impairment, lasting 8–18 days. Therapy with oxaliplatin and UFT®/LV was stopped if functional impairment continued longer than 18 days. UFT®/LV was not extended beyond day 14 of the treatment cycle.

Treatment did not resume until haematological recovery (ANC⩾1500 mm^−3^ and platelets ⩾75 000 mm^−3^) and nonhaematological toxicity resolved to baseline or grade ⩽1 (except alopecia). When therapy restarted, UFT was reduced by 50 mg m^−2^ in case of grade 4 haematological toxicity, or febrile neutropenia and grade 3 nonhaematological toxicity (diarrhoea, stomatitis, nausea/vomiting, skin toxicity). Oxaliplatin was reduced by one dose level for grade 3 or 4 haematological toxicity, febrile neutropenia and paresthesia, lasting more than 18 days or any other grade 4 nonhaematological toxicity.

#### Pretreatment and follow-up evaluation

Within 2 weeks before initiation of study treatment, all patients were assessed by physical examination, complete blood cell, differential white blood cell count, serum biochemistry analysis (sodium, potassium, total protein, calcium, alkaline phosphatase, AST/ALT, total bilirubin, LDH, and creatinine), ECG, chest X-ray, and CT-scan of the abdomen and other sites of the disease when appropriate.

During study treatment, complete differential blood counts with platelets and were determined weekly. Serum chemistries including, liver and renal functions were assessed prior to each cycle. Symptoms, body weight, performance status, physical examination and all adverse events were recorded before each treatment cycle. Adverse events were monitored using NCI-CTC 2.

Tumour assessments were performed every 6 weeks (two cycles) by clinical and CT-scan, employing the same method used to measure the initial targets. Response was determined according to RECIST criteria (Therasse *et al*, 2000). In case of partial or complete response, a second assessment 4 weeks later was required for confirmation of response; all tumour measurements were reviewed and confirmed by an independent panel of radiologists (IRC).

After discontinuation of study treatment, all patients were followed up every 3 months until death. Patients who discontinued treatment for reasons other than progression were evaluated every 3 months for tumour response until progression occurred.

### Statistical methods

#### Study objectives and assessment of response

The primary objective of the study was to characterize the clinical efficacy of the UFT®/LV/oxaliplatin combination treatment by defining objective response rate. This was the proportion of evaluable patients who showed complete and partial responses to treatment.

All patients, who have received at least two cycles of study treatment and have at least one tumour assessment were considered evaluable for response. Secondary objectives were to assess the safety and tolerability of the combination, response duration, time to progression (TTP), and survival time.

#### Sample size and statistical considerations

The study tested the hypothesis that the combination of UFT/LV/oxaliplatin would produce a tumour response rate of >20% in patients with metastatic colorectal cancer. Two-sided Clopper–Pearson confidence intervals for the objective response rates were calculated and adjusted for the two-stage design utilized in the study. The study utilized an optimal two-stage design (Simon, 1989); the first 19 evaluable patients had to demonstrate response to treatment in at least five patients before the second stage could proceed to enter the remaining patients.

Duration of response, survival and time to progression were calculated using the Kaplan–Meier product limit method. Descriptive statistics were utilized in safety analyses and adverse event assessments.

## RESULTS

### Patients characteristics

Between February 2002 and October 2002, 64 patients were enrolled onto this trial at ten French institutions. All received at least one cycle study treatment and were evaluable for safety analysis. Patient characteristics are depicted in Table 1. There were 28 female (44%) and 36 male (56%) with a median age of 68 years (range: 38–82). The median time from initial diagnosis to study entry was 2.6 months (range: 0 days – 155 months). In all, 48 (75%) of patients had synchronous metastasis.

A total of 26 (41%) patients had two or more than two metastatic sites. The most frequent sites involved were liver (47%), lung (9%), liver plus lung (20%), lymph nodes (2%), and the peritoneum (2%). In all, 17 (27%) patients had received previous adjuvant and/or neo-adjuvant chemotherapy, of whom 100% had been treated with an i.v. 5-FU-based regimen.

### Exposure to treatment

A total of 355 cycles were administered with a median number of cycles per patient of six (range 1–14). The median duration of treatment was 18 weeks (range: 3–42 weeks). Dose reduction of UFT® was needed in only 5% of the cycles administered (17/355 cycles), mostly due to gastrointestinal toxicity (3% of the cycles). Dose reduction of oxaliplatin was required in 6% of the cycles, mainly because of neurotoxicity (4% of the cycles). The median value of UFT® and oxaliplatin dose intensity by cycle were 200.0 mg m^−2^ day^−1^±36.8 and 43.3 mg m^−2^ week^−1^±5.1, respectively, and the relative dose intensity by cycle were 100% (range 0–241.4) and 100% (range 0–110.5) for UFT® and oxaliplatin respectively. Reasons for premature study discontinuation (study-withdrawal before cycle 3) were disease progression in six patients, adverse events in three patients, patient request in three patients and death of unknown origin in one patient.

### Independent review committee efficacy results

A total of 58 patients were evaluable for response (Table 2). Five patients were not assessable for response because of early study-discontinuation as a result of early progression (one patient), patient refusal to continue treatment after one cycle (one patient), occurrence of adverse events (three patients) (grade 3 vomiting, grade 3 diarrhoea, and grade 4 anorexia). One additional patient was not evaluable after the IRC evaluation because baseline tumour assessment more than 6 weeks before study entry. A complete response to study treatment was seen in one patient (2%) and a partial response in 19 patients (33%). Thus, the objective tumour response rate in the evaluable patient population was 34% (95% CI: 22–47). In the intent to treat (ITT) population (*n*=64), the objective response rate was 31% (95% CI: 20–43). Stabilization of disease lasting for at least 6 weeks was observed in 29 patients (50%), whereas progressive disease was apparent in nine patients (15%).

**Figure 1:**
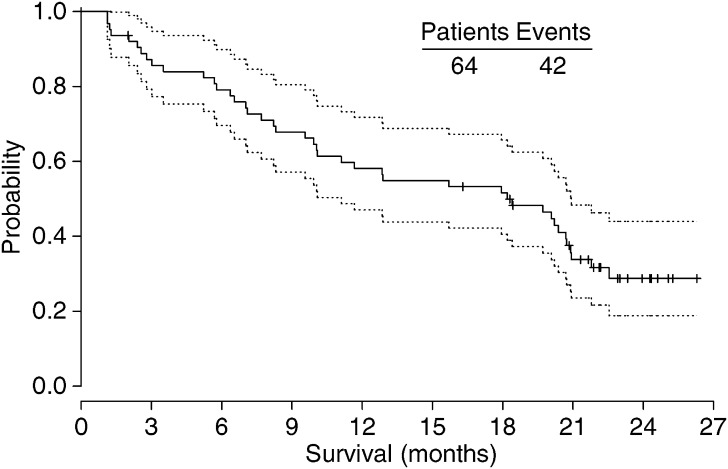
Median Survival – Kaplan–Meier curve.

Analysis of Kaplan–Meier survival curves revealed that the median time to progression (TTP) was 5.88 months (95% confidence intervals: 4.34–8.21months) and the median survival 18.2 months (95% confidence intervals: 10.00–20.70 months) (Figure 1).

### Safety results (Table 3)

The NCI-CTC 2 was used for adverse event reporting and overall treatment tolerance was acceptable (Table 3). Neutropenia was common, but mild to moderate; 35% of patients experienced grade 1 or 2 with only 10% of patients with grade 3 or 4 neutropenia. No patient experienced febrile neutropenia at any grade. Anaemia was graded 3 or 4 in only 6% of patients (grade 1 or 2: 58% of patients). Grade 3 or 4 thrombocytopenia concerned 14% of the patients.

Of the nonhaematological adverse events, gastrointestinal toxicity and asthenia were most commonly reported. In all, 40% of patients experienced grade 1 or 2 diarrhoea (grade 3 or 4: 11% of patients). Other clinically adverse events included grade 3 or 4 nausea/vomiting (11% of the patients), and grade 3 or 4 asthenia (13%). The incidence of stomatitis and hand-foot syndrome were low (14 and 3% of the patients, no grade 3/4). Oxaliplatin-related sensory neuropathy was most commonly evaluated as grade 1 (47% of the patients) or 2 (27% of the patients) while grade 3 and 4 episodes occurred in 13 and 2% of the patients, respectively.

## DISCUSSION

Over the last 20 years, solid progress has been made in chemotherapy for metastatic colorectal cancer. The initial use of 5-FU alone has been replaced by polychemotherapy regimens, such as 5-FU/leucovorin/irinotecan or 5-FU/leucovorin/oxaliplatin, which achieve median survivals of 20 months. After the 1990s, new 5-FU oral prodrugs have been developed providing a better quality of life by suppressing the discomfort of drip-line and possible events related to i.v. injection, such as infection or extravasation. The increasing emphasis on developing oral agents with at least comparable efficacy but enhanced tolerability is an important factor when considering the future care of cancer patients. In addition, when given the choice, patients express a strong preference for oral chemotherapy, provided it is as efficient as standard options intravenous options (Liu *et al*, 1997; Borner *et al*, 2002a).

The safety data that emerged from this study are consistent with previously reported phase I study treating this patient population with UFT®plus LV and with oxaliplatin (Douillard and Seitz, 2001). The toxicity profile of the treatment combination, in the doses and treatment cycles used in this study, was manageable. Most haematological and nonhaematological adverse events were mild or moderate in intensity. Grade 4 NCI-CTC 2 events were rare. The incidence of grade 3/4 sensory neuropathy toxicity was consistent with previously reported studies treating this patient population with 5-FU/LV and oxaliplatin (de Gramont *et al*, 2000).

Many trials have investigated the use of oxaliplatin plus i.v. 5-FU/LV and this combination has become a standard of care for MCRC. In order to avoid the risk and constraints of infusional 5-FU, substitution of oral fluoropyrimidines like UFT for i.v. 5-FU needs to be explored. In the phase I combination trial, the combined used of UFT-folinic acid-and oxaliplatin (TEGAFOX) was tested and the recommended dose for phase II established as UFT 300 mg m^−2^ day^−1^ with leucovorin 90 mg day^−1^ for 14 days, and oxaliplatin 130 mg m^−2^ at day 1 every 3 weeks. UFT/LV in first-line metastatic colorectal cancer has shown similar activity in term of response rate and median survival when compared to Mayo clinic bolus 5-FU/LV regimen (Carmichael *et al*, 2002; Douillard *et al*, 2002). The overall response rate of 34% obtained with this combination of UFT/LV/oxaliplatin is lower than those observed with i.v. 5-FU/LV plus oxaliplatin (45–53%) (de Gramont *et al*, 2000; Giacchetti *et al*, 2000; Goldberg *et al*, 2004). In addition, regimens with capecitabine and oxaliplatin gave objective response rates of 45–49% (Borner *et al*, 2002b; Cassidy *et al*, 2004). However, median survival of 18.2 months reported in our study is comparable to those observed with i.v. 5-FU-LV plus oxaliplatin combinations (around 20 months) or with other regimens testing oxaliplatin with capecitabine (16.8–19.5 months). Another phase II trial was recently published testing the same association with UFT, LV, and oxaliplatin in patients with advanced colorectal cancer (Feliu *et al*, 2004). In all, 84 patients received oxaliplatin on days 1 and 15 (85 mg m^−2^) combined with UFT (390 mg m^−2^ day^−1^) and LV from day 1–14. Results were similar with an objective response rate of 35%, a median time to progression of 7.3 months and a median survival of 16.8 months. A high gastrointestinal toxicity conducted the investigators to reduce the dose of UFT at 300 mg m^−2^ after the inclusion of the first 16 patients. In addition, the combination of UFT-LV and Oxaliplatin was evaluated in two other trials including either patients of 70 years or older (Rosati *et al*, 2005) and patients previously treated with a fluoropyrimidine-based regimen (Kim *et al*, 2002). In elderly patients, the objective response rate was 47% with a median time to disease progression of 8 months (Rosati *et al*, 2005). After failure to fluoropyrimidine-based regimen, the response rate was 12.9% in 31 assessable patients (Kim *et al*, 2002).

The study outcome suggests that the defined combination regimen (UFT/LV/oxaliplatin) is a feasible, safe and convenient outpatient treatment option for patients with nonresectable metastatic colorectal cancer. Efficacy data should be confirmed by randomized phase III. In our study and in the trials conducted by Féliu *et al* and Rosati *et al* (2005) the schedule of oxaliplatin administration was different: 130 mg m^−2^ every 21 days in our study and 85 mg m^−2^ at days 1 and 14 (Feliu *et al*, 2004) or 65 mg m^−2^ at days 1 and 8. Efficacy of these three schedules is probably similar but our schedule should be preferred for the comfort of patients, reducing the frequency of i.v. administration. Better understanding of tumour cell biology has actively contributed to the development of novel biological agents that block certain cell growth events. These new strategies, particularly inhibition of angiogenesis and Epidermal Growth Factor receptors, can be used in association with chemotherapy to increase survival.

In the future, we can expect that oral 5-FU prodrugs will replace i.v. 5-FU in combination with oxaliplatin or irinotecan. The role of additional agents, such as anti-EGFR compounds or angiogenesis inhibitors should be defined in the therapeutic strategy of the metastatic colorectal cancers, in combination with oral fluoropyrimmidine containing regimen.

## Figures and Tables

**Table 1:** Patient and disease characteristics at baseline (*n*=64)

Number of patients	64
Male/female (% of patients)	36 (56%)/28(44%)
Median age (range)	68 years (38–82)
	
*Karnofsky performance status (KPS)*	
0	40 (63%)
1	24 (37%)
	
*Primary disease*	
Colon	49 (77%)
Rectal	15 (23%)
	
*Number of metastatic sites*	
1	38 (59%)
2	21 (33%)
>2	5 (8%)
	
*Sites of metastasis*	
Liver	30 (47%)
Lung	6 (9%)
Liver+lung	13 (20%)
Lymph nodes	1 (2%)
Peritoneum	1 (2%)
Others	13 (20%)
	
*Prior adjuvant chemotherapy (5-FU based regimen)*	
Yes/no	17(27%)/47(73%)

**Table 2:** Response rates (IRC assessment)

	**Evaluable population (*N*)**	**Intent to treat population (*N*)**
Overall	58 (91%)	64 (100%)
Not assessable		6 (9%)
Complete response (CR)	1 (2%)	1 (2%)
Partial response (PR)	19 (33%)	19 (30%)
Stable disease (SD)	29 (50%)	29 (45 %)
Progressive disease (PD)	9 (15%)	9 (14 %)
		
ORR	20 (34%)	20 (31%)
95% CI	(22–47)	(20–43)

**Table 3:** Toxicity by patient and by cycle (NCI/CTC grade)

	**By patient**		**By cycle**	
	**All grades (%)**	**Grade3–4 (%)**	**All grades (%)**	**Grade3–4 (%)**
Leucopenia	41	0	21	0
Neutropenia	45	10	23	2
Anaemia	58	6	30	2
Thrombocytopenia	44	14	18	4
Infection	28	10	5	1
				
*Gastrointestinal*				
Nausea	66	5	33	1
Vomiting	42	6	25	1
Diarrhoea	51	11	22	2
Stomatitis	14	0	6	0
				
Hand-foot syndrome	3	0	1	0
Sensory neuropathy	89	15	79	3
Asthenia	74	13	33	2
Alopecia	5	0	16	0
